# Clinical efficacy of tofacitinib in active ankylosing spondylitis and dynamic changes in gut microbiota: an exploratory descriptive four-case series with 16S rRNA sequencing analysis

**DOI:** 10.3389/fimmu.2026.1784400

**Published:** 2026-03-31

**Authors:** Lin Wang, Weilin Xie, Jiaoyan Li, Junjie Chen, Yinghong Huang

**Affiliations:** 1 Department of Rheumatology and Nephrology, The First Hospital of Changsha, Changsha, China; 2 Military Treatment Center, The 970th Hospital of the People’s Liberation Army Joint Logistics Support Force, Yantai, China

**Keywords:** ankylosing spondylitis, Janus kinase inhibitor, tofacitinib, gut microbiota, 16S rRNA sequencing

## Abstract

**Objective:**

To describe short-term clinical outcomes and safety of tofacitinib in four patients with active ankylosing spondylitis (AS) using real-world data, and to explore dynamic changes in gut microbiota before and after treatment.

**Methods:**

Four patients with active AS received oral tofacitinib 5 mg twice daily for 6 months. Disease activity [the bath ankylosing spondylitis disease activity index (BASDAI), the ankylosing spondylitis disease activity score calculated with C-reactive protein (ASDAS-CRP)], functional status [the bath ankylosing spondylitis functional index (BASFI)], back pain visual analog scale (VAS), inflammatory markers [CRP, erythrocyte sedimentation rate (ESR)], and adverse events were assessed at baseline and month 6. Paired stool samples were collected for 16S ribosomal ribonucleic acid (rRNA) sequencing to evaluate alpha/beta diversity and compositional changes.

**Results:**

After 6 months, all four patients showed marked improvement in disease activity and function: BASDAI decreased from 8.38 ± 0.52 to 2.60 ± 0.38; ASDAS-CRP from 3.85 ± 0.29 to 1.50 ± 0.26. CRP and ESR trended downward, and chest expansion increased. No serious adverse events were observed. Microbiota analysis revealed no consistent changes in alpha diversity or beta diversity clustering; however, individual-level shifts in genera such as Faecalibacterium, Alistipes, and Acidaminococcus were observed.

**Conclusion:**

In this exploratory descriptive case series, tofacitinib was associated with favorable short-term clinical outcomes in four patients with active AS. No serious adverse events were noted. Gut microbiota alterations were highly individualized and hypothesis-generating only. Larger controlled studies are warranted to explore potential gut–joint mechanisms.

## 1 Introduction

Ankylosing spondylitis (AS) is the classic radiographic subtype of axial spondyloarthritis (axSpA), typically onset in young adults. It is characterized by chronic inflammatory back pain, morning stiffness, and inflammation of the spine and sacroiliac joints, which can lead to functional limitation, spinal deformity, and significant decline in quality of life. In the Chinese population, the prevalence of AS is approximately 0.20%~0.42%, with a high human leukocyte antigen (HLA)-B27 positivity rate of 88.8%~89.4%, of which HLA-B*2704 is the most common subtype (1). AS patients often exhibit elevated inflammatory markers, and disease activity correlates positively with markers such as C-reactive protein (CRP) and erythrocyte sedimentation rate (ESR) (2). Studies have shown that serum pro-inflammatory cytokines such as interleukin (IL)-6 and IL-1β levels are associated with the clinical response to non-steroidal anti-inflammatory drugs (e.g., celecoxib) in AS patients, suggesting a key role for cytokine networks in disease activity and treatment response (3).

Regarding treatment strategies, non-steroidal anti-inflammatory drugs (NSAIDs) remain first-line foundational therapy. When NSAIDs are insufficient or in cases of high disease activity, the updated 2022 Assessment of SpondyloArthritis international Society-European Alliance of Associations for Rheumatology (ASAS-EULAR) recommendations suggest further use of tumor necrosis factor (TNF) inhibitors, IL-17 inhibitors, or targeted synthetic disease-modifying antirheumatic drugs (DMARDs) (e.g., Janus kinase inhibitors (JAKi)) to control inflammation and symptoms (4). Tofacitinib is an oral JAKi that has demonstrated significant improvement in AS disease activity indices in randomized controlled trials, with an overall acceptable safety profile during follow-up (5, 6). However, real-world evidence for tofacitinib in AS and its underlying biological mechanisms require further accumulation.

In recent years, the “gut-joint axis” has been recognized as an important link in the pathogenesis and persistent inflammation of AS. Several quantitative metagenomic studies have revealed unique gut microbiome biomarkers in AS patients, characterized by increased abundance of Prevotella species such as Prevotella copri and P. melaninogenica, and decreased abundance of Bacteroides (7). This dysbiosis may be involved in the pathogenesis of AS. Furthermore, the gut microbiomes of AS and inflammatory bowel disease (IBD) show significant differences, suggesting potentially distinct roles of the gut in driving these two diseases (8). However, the impact of biologics or small molecule targeted drugs on the gut microecology and its relationship with efficacy remains inconclusive, and current evidence is largely descriptive (9). For example, anti-TNF-α therapy can partially restore gut microbiota dysbiosis in AS patients, particularly by increasing the abundance of short-chain fatty acid-producing bacteria (e.g., Megamonas), which correlates with reduced disease activity (10). Additionally, immune cells, especially macrophages, play a crucial role in the inflammatory process of AS. Studies have shown that macrophages from AS patients release higher levels of TNF-α and IL-1β under inflammatory stimulation, which may be a key mechanism for persistent disease activity (11).

The janus kinase-signal transducer and activator of transcription (JAK-STAT) signaling pathway plays a pivotal role in mucosal immune regulation and epithelial barrier homeostasis. *In vitro* studies have shown that tofacitinib can ameliorate inflammatory cytokine-induced intestinal epithelial barrier dysfunction (12), suggesting a hypothetical involvement in disease modulation through the “mucosal immunity-barrier-microbiota” network. This hypothesis requires direct functional validation.

Considering the central role of the IL-23/IL-17 axis in AS pathogenesis (13) and the potential inhibitory effect of JAK inhibitors on downstream signaling of this pathway, tofacitinib may influence the gut immune microenvironment and microbiota homeostasis by regulating this key inflammatory axis. Based on this, this study reports the clinical outcomes of four patients with active AS treated with tofacitinib for 6 months, combined with paired stool 16S ribosomal ribonucleic acid (rRNA) sequencing to describe changes in gut microbiota before and after treatment, aiming to provide preliminary, hypothesis-generating real-world evidence.

## 2 Materials and methods

### 2.1 Study subjects

This was a retrospective exploratory descriptive case series. Four consecutive patients with active AS who attended the Department of Rheumatology and Nephrology, The First Hospital of Changsha, were included. Inclusion criteria: meeting the 1984 modified New York criteria; moderate to high disease activity at baseline (Bath Ankylosing Spondylitis Disease Activity Index (BASDAI) ≥4 and/or Ankylosing Spondylitis Disease Activity Score (ASDAS) ≥2.1); inadequate response to NSAIDs; and discontinuation of biologics or targeted synthetic DMARDs for at least 6 months prior to enrollment. All participants confirmed no use of antibiotics, probiotics, or prebiotics within 3 months before stool sampling. Dietary intake was not standardized prior to sampling, which is acknowledged as a limitation.

### 2.2 Treatment and follow-up

All patients received oral tofacitinib 5 mg twice daily for 6 months. Concomitant NSAIDs (e.g., celecoxib 200 mg/day or adjusted as needed) were permitted. During follow-up, safety assessments and laboratory monitoring (complete blood count, liver/kidney function, lipids, etc.) were performed according to clinical routine. Risk assessments included screening for latent tuberculosis, hepatitis B/C, and other infections. Adherence, concomitant medications, and dropouts were recorded. Lipid profiles were monitored at baseline and month 6. No clinically significant lipid elevations were observed.

### 2.3 Clinical assessment

Disease activity and function indices (BASDAI, BASFI, ASDAS), back pain VAS, and chest expansion were assessed at baseline (M0) and month 6 (M6). ESR and CRP were measured. Treatment response was defined according to ASAS/ASDAS criteria (e.g., ASDAS <2.1 for low disease activity, <1.3 for clinical remission). Patient Global Assessment (PGA) and Physician Global Assessment (PhGA) were also recorded. ASDAS-CRP thresholds for clinically important improvement, major improvement, and inactive disease were defined according to ASAS recommendations. ASAS20/ASAS40 response rates were calculated and are reported. Chest expansion was measured using a non-elastic tape measure at the level of the fourth intercostal space. The difference in chest circumference between maximum inspiration and maximum expiration was recorded, and the best value of three measurements was used.

### 2.4 Stool sample collection and 16S rRNA sequencing

Morning stool samples were collected at baseline (M0) and month 6 (M6) (time of day was not strictly standardized, acknowledged as a limitation), stored in sterile cryotubes at -80 °C, and sent for analysis. Total DNA was extracted, the V4 region of the 16S rRNA gene was amplified, and high-throughput sequencing was performed. Raw sequences underwent quality control, denoising, Amplicon Sequence Variant (ASV) construction, and taxonomic annotation for subsequent diversity and compositional analysis. Bioinformatics analysis was performed using QIIME2 or similar pipelines.

### 2.5 Bioinformatics and statistical analysis

Given the small sample size (n = 4), the analysis was primarily descriptive. Alpha diversity was assessed using Chao1 and Shannon indices, and beta diversity was evaluated via Bray–Curtis distance and visualized with principal coordinates analysis (PCoA). Paired comparisons were performed using the Wilcoxon signed-rank test, but due to the limited sample size, P values are not emphasized and should be interpreted with caution. All analyses were conducted using R software.

## 3 Results

### 3.1 Baseline patient characteristics

The cohort consisted of three males and one female, with a mean age of 38.5 years (range 31–50) and a disease duration ranging from 3 to 18 years. All patients were HLA-B27 positive. Comorbidities and Extra-Articular Manifestations: One patient had a history of hypertension and recurrent uveitis. The remaining three patients had no reported comorbidities or extra-articular involvement. Lifestyle Factors: Lifestyle habits varied considerably: two patients maintained a balanced diet and a regular daily routine. Prior Treatment History: All patients had previously been treated with conventional synthetic DMARDs (csDMARDs) and NSAIDs. Three patients had previously received biologic therapy, including recombinant human TNF receptor or infliximab, all discontinued at least six months prior to enrollment. Baseline Disease Activity: At baseline, all patients presented with high disease activity, with a mean BASDAI of 8.38 and a mean ASDAS-CRP of 3.85. Inflammatory markers (CRP and ESR) were elevated across the cohort. In summary, despite uniformly high disease activity at baseline, the four cases exhibited considerable heterogeneity in terms of disease duration, extra-articular manifestations, prior treatment exposure, and lifestyle habits—factors that may influence both clinical response and gut microbiota composition (**Table 1**).

**Table 1:** Baseline characteristics of four patients with active AS.

Characteristic	Case X	Case J	Case G	Case T
Age (years)/Sex	35/F	31/M	50/M	38/M
Disease duration (years)	3	5	18	14
HLA-B27	Positive	Positive	Positive	Positive
Smoking history	None	Yes (10 years, quit 2 years ago)	Yes (20 years, current)	None
Alcohol consumption	None	Occasional	3–4 times/week	None
BMI (kg/m²)	22.3	24.1	26.5	23.8
Comorbidities	None	None	Hypertension (Grade 1)	None
Extra-articular manifestations	None	None	Recurrent uveitis (2 episodes)	None
Prior biologic/JAKi therapy	Recombinant human type II TNF receptor (6 months)	Adalimumab (5 months)	Infliximab (6 months)	Adalimumab (6 months)
Prior conventional DMARDs/NSAIDs	Meloxicam, sulfasalazine	Celecoxib, thalidomide, sulfasalazine	Prednisone, celecoxib, thalidomide	Celecoxib, thalidomide, sulfasalazine
Lifestyle factors (diet/activity)	Balanced diet, regular routine	High-fat diet, late nights	High-sugar diet, sedentary, irregular routine	Balanced diet, regular routine
Antibiotics/probiotics use within 3 months	None	None	None	None
Baseline BASDAI	8.5	9.0	7.8	8.2
Baseline BASFI	7.8	8.5	7.2	7.9
Baseline CRP (mg/L)	28.5	32.0	24.8	26.3
Baseline ESR (mm/h)	45	52	38	41
Baseline ASDAS-CRP	3.9	4.1	3.6	3.8
Baseline back pain VAS (0–10)	8	9	7	8
Chest expansion (cm)	2.0	2.5	2.8	2.0

AS, Ankylosing Spondylitis; F, Female; M, Male; BASDAI, Bath Ankylosing Spondylitis Disease Activity Index; BASFI, Bath Ankylosing Spondylitis Functional Index; ASDAS, Ankylosing Spondylitis Disease Activity Score; CRP, C-reactive protein; ESR, Erythrocyte sedimentation rate.

### 3.2 Clinical efficacy and safety

After 6 months of treatment, all four patients showed marked improvements in disease activity, function, and inflammatory markers compared to baseline (**Table 2**). BASDAI decreased from 8.38 ± 0.52 to 2.60 ± 0.38; BASFI from 7.85 ± 0.55 to 2.38 ± 0.37; ASDAS from 3.85 ± 0.29 to 1.50 ± 0.26; and back pain VAS from 8.00 ± 0.82 to 2.25 ± 0.96. Inflammatory markers CRP and ESR showed a decreasing trend. Chest expansion increased from 2.33 ± 0.42 cm to 3.73 ± 0.44 cm.

**Table 2:** Changes in clinical and laboratory indicators before and after tofacitinib treatment (n=4).

Indicator	Baseline (M0) Mean ± SD	Month 6 (M6) Mean ± SD	Change (Δ) Mean ± SD
BASDAI	8.38 ± 0.52	2.60 ± 0.38	−5.78 ± 0.47
BASFI	7.85 ± 0.55	2.38 ± 0.37	−5.48 ± 0.51
ASDAS	3.85 ± 0.29	1.50 ± 0.26	−2.35 ± 0.25
VAS Back Pain (score)	8.00 ± 0.82	2.25 ± 0.96	−5.75 ± 0.96
CRP (mg/L)	31.14 ± 24.32	11.18 ± 13.93	−19.96 ± 10.49
ESR (mm/h)	50.15 ± 37.32	12.70 ± 8.02	−37.45 ± 29.31
Chest Expansion (cm)	2.33 ± 0.42	3.73 ± 0.44	+1.40 ± 0.08

Data are presented as mean ± standard deviation; Change (Δ) = Post-treatment – Pre-treatment; BASDAI, Bath Ankylosing Spondylitis Disease Activity Index; BASFI, Bath Ankylosing Spondylitis Functional Index; ASDAS, Ankylosing Spondylitis Disease Activity Score; CRP, C-reactive protein; ESR, Erythrocyte sedimentation rate; VAS, Visual Analog Scale. Data are presented descriptively. Due to the very small sample size (n=4), inferential statistics were not applied and P values are not reported. Individual patient trajectories are available upon request.

ASAS20 response was achieved in 4/4 patients; ASAS40 response was achieved in 3/4 patients. All patients tolerated the treatment well throughout follow-up. No serious infections, thrombotic events, other serious adverse events requiring drug discontinuation, or clinically significant laboratory abnormalities (including lipid elevations) were observed.

### 3.3 Gut microbiota diversity changes

**Figure 1:**
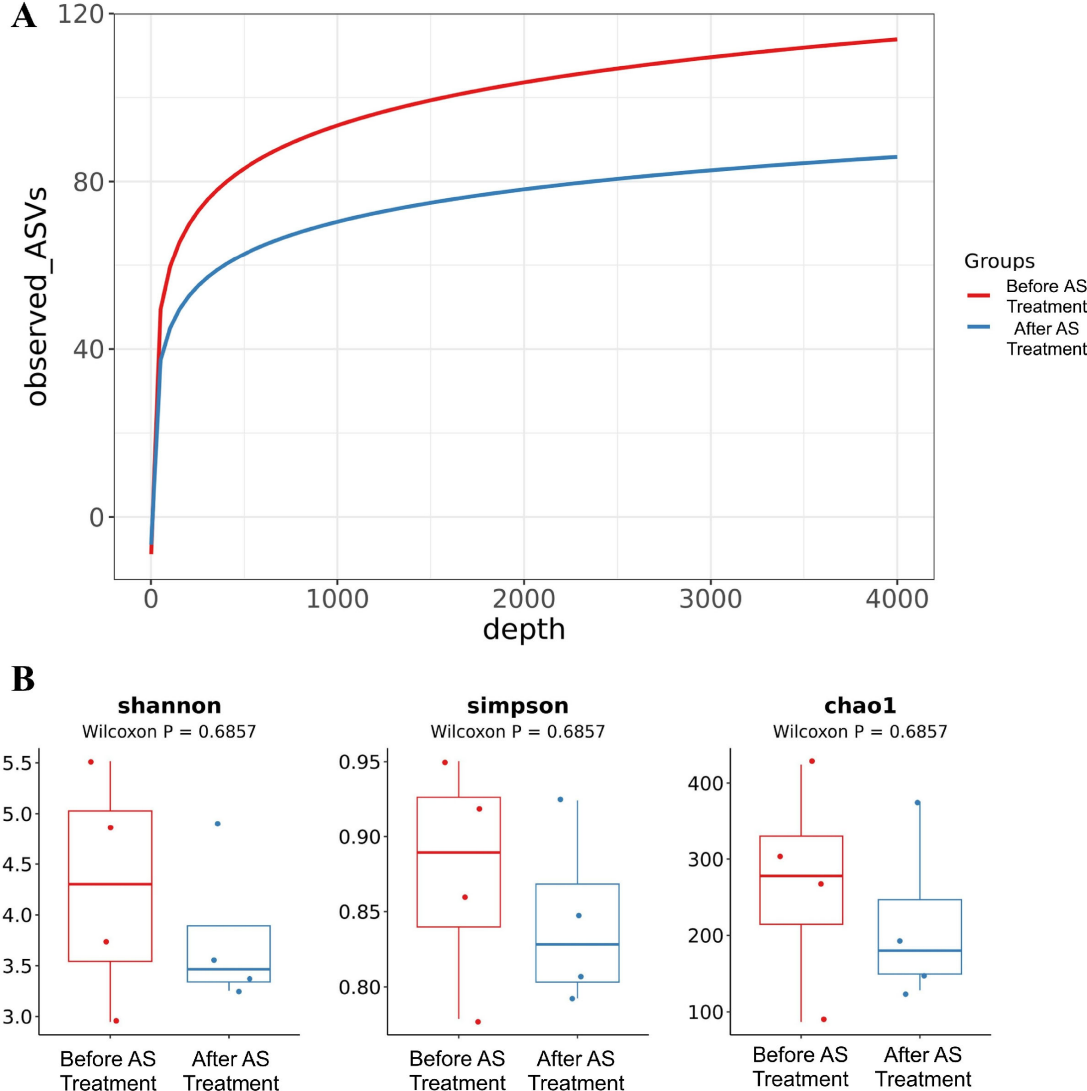
Comparison of sequencing depth and alpha diversity of fecal microbiota before and after tofacitinib treatment. **(A)** Rarefaction curves: X-axis represents sequencing depth, Y-axis represents Observed ASVs. Red lines indicate pre-treatment AS samples, blue lines indicate post-treatment AS samples. The plateauing curves suggest sequencing depth was sufficient to cover sample diversity. **(B)** Boxplots of alpha diversity indices: Comparison of Shannon index, Simpson index, and Chao1 index between pre- and post-treatment groups. Dots represent individual samples; boxplots show distribution. Due to the small sample size, statistical comparisons are descriptive and should not be overinterpreted. P values are not shown.

A total of 8 qualified stool 16S sequencing datasets were obtained (4 pre-treatment, 4 post-treatment). Sequencing depth was relatively balanced across samples, with valid sequence proportions ranging from 96.27% to 99.46%. Rarefaction curves showed that Observed ASVs gradually reached a plateau with increasing sequencing depth, indicating that the sequencing depth was sufficient to cover sample microbial diversity (**Figure 1A**).

In alpha diversity analysis, no consistent directional changes were observed in Shannon and Chao1 indices before and after treatment (**Figure 1B**).

### 3.4 Paired changes in key bacterial genera

**Figure 2:**
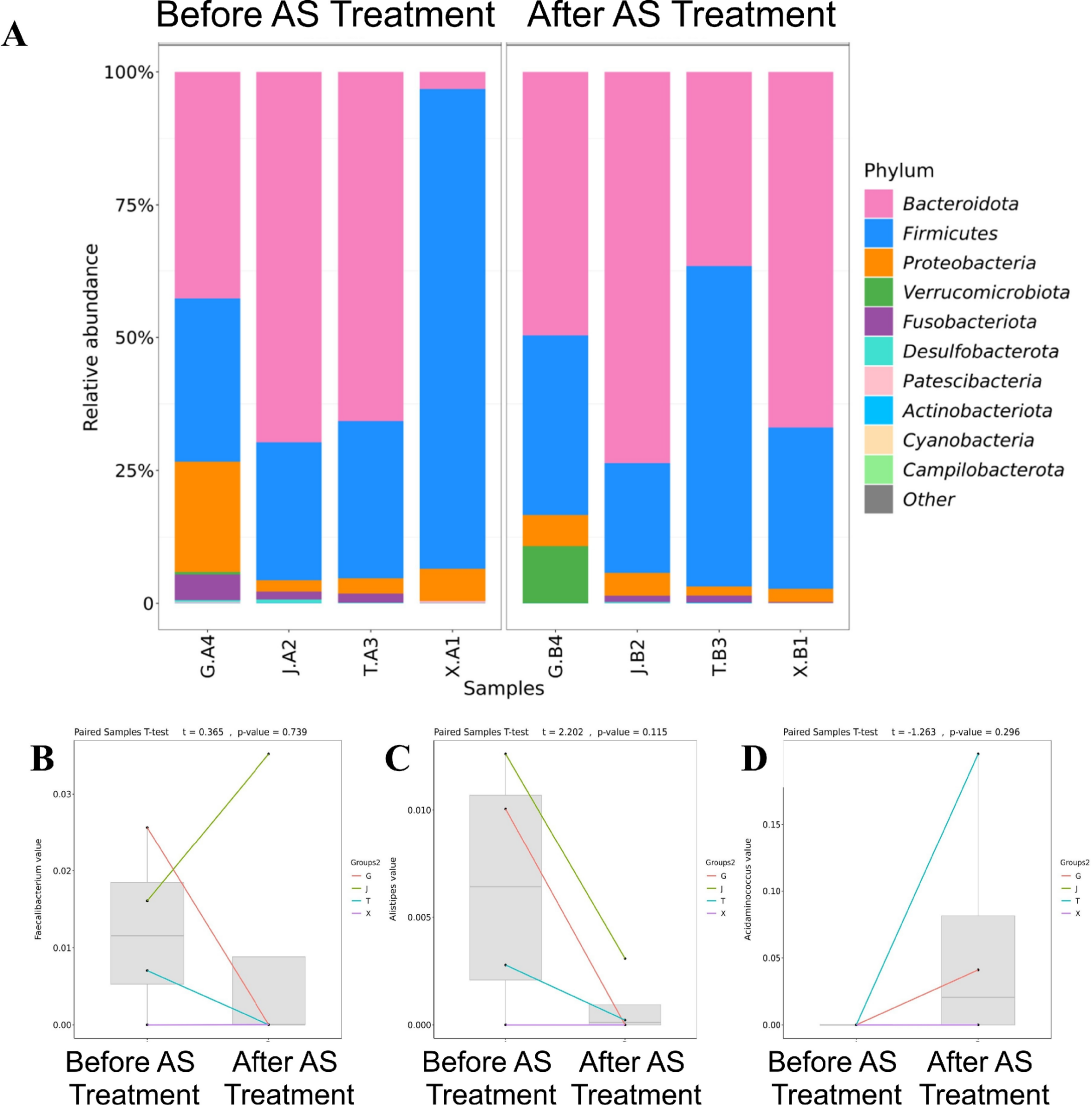
Gut microbiota composition and paired changes of key genera before and after AS treatment. **(A)** Stacked bar charts showing relative abundance at the phylum level in fecal microbiota from four subjects (G, J, T, X) pre-treatment (sample code “A”, e.g., G.A4, J.A2, T.A3, X.A1) and post-treatment (sample code “B”, e.g., G.B4, J.B2, T.B3, X.B1). **(B–D)** Paired changes in relative abundance of key genera pre- and post-treatment: **(B)** Faecalibacterium, **(C)** Akkermansia, **(D)** Streptococcus. Lines connect paired samples from the same subject; gray boxplots summarize intra-group distribution.

Despite the lack of prominent overall diversity changes, “individualized remodeling” of microbiota composition was observed in paired individual comparisons. At the phylum level, Bacteroidota and Firmicutes remained the dominant phyla in fecal microbiota before and after treatment, with no consistent directional shift in overall composition; inter-individual differences were more pronounced than pre-post treatment differences, with fluctuations in the relative abundance of phyla like Proteobacteria observed in some cases (**Figure 2A**). In paired comparisons at the genus level, no consistent directional shifts were observed in Faecalibacterium, Akkermansia, or Streptococcus across individuals (14) (**Figure 2B**). Paired t-tests were not applied due to small sample size and violation of normality assumptions. These findings are hypothesis-generating only and require validation in larger cohorts.

Akkermansia showed a decreasing trend in most cases (**Figure 2C**); Streptococcus had low overall abundance pre-treatment, increased in some individuals post-treatment (**Figure 2D**). Notably, potentially pathogenic genera like Streptococcus and Haemophilus have been reported to be enriched in AS and IBD patients and associated with disease activity (8, 15). Therefore, despite the lack of prominent changes at the aggregate level, “individualized remodeling” of microbiota composition was evident in paired individuals. Due to the small sample size (n=4 pairs), these differences are insufficient for robust statistical inference and require validation in larger samples to further explore their correspondence with decreasing inflammatory markers and clinical remission.

## 4 Discussion

Based on real-world cases of four patients with active AS, this exploratory descriptive study observed marked improvements in BASDAI, BASFI, ASDAS-CRP, and back pain VAS after 6 months of tofacitinib treatment, along with an increase in chest expansion. These preliminary findings are consistent with prior randomized controlled trial results (5, 6). However, due to the uncontrolled design and small sample size, causality cannot be inferred. As highlighted in recent reviews of study design in rheumatology, uncontrolled case series are valuable for generating hypotheses but are inherently susceptible to bias. Alternative explanations-–including regression to the mean, natural disease fluctuation, placebo effects, and concomitant NSAID use-–cannot be excluded.

While the observed clinical improvement is promising, its underlying mechanisms remain to be elucidated. The clinical benefits may be related to tofacitinib’s known role as a broad-spectrum inhibitor of the JAK-STAT signaling pathway, which modulates multiple pro-inflammatory cytokines implicated in AS pathogenesis, including IL-6 and those of the IL-23/IL-17 axis (3) (13). Prior studies have confirmed that serum levels of cytokines like IL-6 are independently associated with treatment response in AS patients (3). However, attributing the observed improvements specifically to these pathways would be speculative based on the current data.

In previous randomized controlled trials, tofacitinib significantly increased ASAS20/40 response rates and improved BASDAI and ASDAS indices, with an overall manageable adverse event profile (5). Recent integrated safety analyses indicated low incidence rates of herpes zoster, serious infections, and major adverse cardiovascular events in the AS population, although monitoring remains necessary (6). In this limited cohort, no serious adverse events were observed during short-term follow-up. It is crucial to emphasize that long-term safety and the detection of rare adverse events cannot be assessed from this small case series.

Beyond symptom control, the pathological process of AS is hypothesized to be linked to gut microecology and mucosal immunity-a concept known as the “gut-joint axis.” Systematic reviews suggest decreased microbiota diversity and compositional differences in AS patients, but with marked heterogeneity between studies, potentially related to ethnicity, diet, medication, and analytical methods (9). In this context, our study did not observe consistent changes in alpha or beta diversity of the gut microbiota following tofacitinib treatment. This finding contrasts with some studies of anti-TNF-α therapy, which have shown a partial restoration of gut microbiota dysbiosis in AS patients (10). The lack of consistent shifts in our study may suggest that JAK inhibitors have a limited or more variable impact on the overall structure of gut microbiota, or that a longer observation period is needed to detect changes.

The highly individualized nature of the microbiota changes observed in our four patients warrants careful interpretation. The inter-individual variability may be related to factors such as baseline microbiota state, genetic background (e.g., HLA-B27), and prior treatment history (8, 9). While previous multi-omics and Mendelian randomization studies have suggested potential links between specific gut microbes and AS risk (16), our descriptive 16S rRNA data do not permit any conclusions about functional alterations or causal relationships. Therefore, these findings should be viewed strictly as hypothesis-generating, providing a basis for future research rather than evidence of a drug-microbiome mechanism.

This study has several important limitations that preclude definitive conclusions: (1) the small sample size (n = 4) limits generalizability and increases the risk of observing spurious findings; (2) the lack of a control group means that the observed improvements cannot be causally attributed to tofacitinib; (3) potential confounding by uncontrolled variables such as concomitant NSAID use, diet, and undisclosed antibiotic or probiotic use is a major concern; (4) the short follow-up (6 months) precludes assessment of long-term safety and radiographic progression; (5) 16S rRNA sequencing provides taxonomic but not functional information about the microbiota; (6) stool collection was not standardized for diet or time of day, introducing potential pre-analytical variability. These limitations mean that all microbiota findings are descriptive and hypothesis-generating only, and they underscore the need for larger, well-controlled studies to explore the potential role of the gut–joint axis in JAK inhibitor therapy for AS.

## 5 Conclusion

In this exploratory case series, tofacitinib was associated with marked short-term clinical improvement in four patients with active AS, and no serious adverse events were observed. Gut microbiota alterations were highly individualized and inconsistent, precluding any definitive conclusions regarding drug–microbiome interactions. Larger, controlled studies incorporating multi-omics approaches are needed to investigate the potential role of the gut–joint axis in JAK inhibitor therapy for AS.

## Data Availability

The original contributions presented in the study are included in the article/supplementary material. Further inquiries can be directed to the corresponding author.

## References

[1] Zhang S Peng L Li Q Zhao J Xu D Zhao J . Spectrum of spondyloarthritis among chinese populations. Curr Rheumatol Rep. (2022) 24:247–58. doi: 10.1007/s11926-022-01079-1, PMID: 35829981 PMC9307523

[2] Karoli Y Avasthi S Mahapatra S Karoli R . Clinical profile of ankylosing spondylitis at a teaching hospital. Ann Afr Med. (2022) 21:204–7. doi: 10.4103/aam.aam_103_20, PMID: 36204904 PMC9671186

[3] Zhang Y Ning C Zhou H Yan Y Liu F Huang Y . Interleukin-1beta, interleukin-6, and interleukin-17A as indicators reflecting clinical response to celecoxib in ankylosing spondylitis patients. Ir J Med Sci. (2021) 190:631–8. doi: 10.1007/s11845-020-02366-5, PMID: 32955700

[4] Ramiro S Nikiphorou E Sepriano A Ortolan A Webers C Baraliakos X . ASAS-EULAR recommendations for the management of axial spondyloarthritis: 2022 update. Ann Rheum Dis. (2023) 82:19–34. doi: 10.1136/ard-2022-223296, PMID: 36270658

[5] Deodhar A Sliwinska-Stanczyk P Xu H Baraliakos X Gensler LS Fleishaker D . Tofacitinib for the treatment of ankylosing spondylitis: a phase III, randomised, double-blind, placebo-controlled study. Ann Rheum Dis. (2021) 80:1004–13. doi: 10.1136/annrheumdis-2020-219601, PMID: 33906853 PMC8292568

[6] Deodhar A Akar S Curtis JR El-Zorkany B Magrey M Wang C . Integrated safety analysis of tofacitinib from Phase 2 and 3 trials of patients with ankylosing spondylitis. Adv Rheumatol. (2024) 64:87. doi: 10.1186/s42358-024-00402-x, PMID: 39695887 PMC13488738

[7] Wen C Zheng Z Shao T Liu L Xie Z Le Chatelier E . Quantitative metagenomics reveals unique gut microbiome biomarkers in ankylosing spondylitis. Genome Biol. (2017) 18:142. doi: 10.1186/s13059-017-1271-6, PMID: 28750650 PMC5530561

[8] Sternes PR Brett L Phipps J Ciccia F Kenna T de Guzman E . Distinctive gut microbiomes of ankylosing spondylitis and inflammatory bowel disease patients suggest differing roles in pathogenesis and correlate with disease activity. Arthritis Res Ther. (2022) 24:163. doi: 10.1186/s13075-022-02853-3, PMID: 35794662 PMC9261041

[9] Su QY Zhang Y Qiao D Song X Shi Y Wang Z . Gut microbiota dysbiosis in ankylosing spondylitis: a systematic review and meta-analysis. Front Cell Infect Microbiol. (2024) 14:1376525. doi: 10.3389/fcimb.2024.1376525, PMID: 39421642 PMC11484232

[10] Dai Q Xia X He C Huang Y Chen Y Wu Y . Association of anti-TNF-alpha treatment with gut microbiota of patients with ankylosing spondylitis. Pharmacogenet Genomics. (2022) 32:247–56. doi: 10.1097/FPC.0000000000000468, PMID: 35852868 PMC9351697

[11] Akhtari M Zargar SJ Vojdanian M Jamshidi A Mahmoudi M . Monocyte-derived and M1 macrophages from ankylosing spondylitis patients released higher TNF-alpha and expressed more IL1B in response to BzATP than macrophages from healthy subjects. Sci Rep. (2021) 11:17842. doi: 10.1038/s41598-021-96262-2, PMID: 34497300 PMC8426480

[12] Spalinger MR Sayoc-Becerra A Ordookhanian C Canale V Santos AN King SJ . The JAK inhibitor tofacitinib rescues intestinal barrier defects caused by disrupted epithelial-macrophage interactions. J Crohns Colitis. (2021) 15:471–84. doi: 10.1093/ecco-jcc/jjaa182, PMID: 32909045 PMC7944512

[13] Deveci H Turk AC Ozmen ZC Demir AK Say Coskun SU . Biological and genetic evaluation of IL-23/IL-17 pathway in ankylosing spondylitis patients. Cent Eur J Immunol. (2019) 44:433–9. doi: 10.5114/ceji.2019.92805, PMID: 32140056 PMC7050057

[14] Sokol H Pigneur B Watterlot L Lakhdari O Bermudez-Humaran LG Gratadoux JJ . Faecalibacterium prausnitzii is an anti-inflammatory commensal bacterium identified by gut microbiota analysis of Crohn disease patients. Proc Natl Acad Sci U S A. (2008) 105:16731–6. doi: 10.1073/pnas.0804812105, PMID: 18936492 PMC2575488

[15] Costello ME Ciccia F Willner D Warrington N Robinson PC Gardiner B . Brief report: intestinal dysbiosis in ankylosing spondylitis. Arthritis Rheumatol. (2015) 67:686–91. doi: 10.1002/art.38967, PMID: 25417597

[16] Du X Li H Zhao H Cui S Sun X Tan X . Causal relationship between gut microbiota and ankylosing spondylitis and potential mediating role of inflammatory cytokines: A mendelian randomization study. PloS One. (2024) 19:e0306792. doi: 10.1371/journal.pone.0306792, PMID: 39083521 PMC11290680

